# Refractory high entropy alloy dataset with room temperature ductility screening

**DOI:** 10.1016/j.dib.2022.108582

**Published:** 2022-09-11

**Authors:** Andrew Detor, Scott Oppenheimer, Rebecca Casey, Cole Crawford

**Affiliations:** GE Research, 1 Research Circle, Niskayuna, NY 12309, United States

**Keywords:** High-throughput, Hardness, Solidus, Arc melting, Ultrahigh temperature metal, Complex concentrated alloy, Multiobjective optimization

## Abstract

This dataset contains 82 unique refractory alloys experimentally synthesized via arc melting and subject to screening tests for hardness and room temperature ductility. Most compositions fall under the definition of high entropy or complex concentrated alloys, but simpler ternary compositions are also included. Hardness was collected via a standard indentation technique while compressive ductility was quantified using a custom high throughput experimental approach yielding a ductility ranking from 0 to 5. The unique ductility screening test was developed to provide directional information for alloy development at a low cost and rapid pace using conventional test equipment. Predicted solidus temperature for all alloys is also included based on thermodynamic modelling. The dataset should be of interest to those exploring the emerging class of refractory high entropy alloys and particularly useful where optimization is sought balancing strength, ductility, and high melting temperature.

**Specifications Table**

**Table:** 

Subject	Metals and alloys
Specific subject area	Refractory high entropy alloys (RHEA) and complex concentrated alloys (CCA).
Type of data	Table (.csv)
How the data were acquired	Alloy test specimens were produced by arc melting from raw elemental stock. Room temperature microhardness was measured on mounted and polished cross sections of each specimen using a standard Vickers indenter (Buehler Micromet 2100) at an applied load of 500g. Separate sections cut from arc melted samples were used to assess room temperature compressive ductility. Sections were compressed in a standard servo-hydraulic test frame (MTS Landmark) at 2.5mm/s displacement rate (corresponding to an initial strain rate on the order of 1 × 10^−3^ s^−1^) with an integrated video camera to observe and rank deformation behavior on a scale from 0-5. Solidus (melting temperature) was estimated using Thermo-Calc software (TCHEA4 database operated through TC-Python).
Data format	Raw
Description of data collection	Alloys containing 3 or more of the following elements are included in this dataset: Hf, Mo, Nb, Re, Ru, Ta, Ti, W, and Zr. The maximum concentration of any single element is 50 atomic percent. All samples were tested in the as-arc melted condition; no subsequent heat treatments were applied. Room temperature compressive ductility ranking (scale from 0-5) was determined using a video standard approach based on the observed deformation behavior of each sample in compression.
Data source location	• Institution: GE Research • City/Town/Region: Niskayuna, NY • Country: USA • Latitude and longitude: 42.829388,-73.873059
Data accessibility	Repository name: Figshare Data identification number: n/a Direct URL to data: https://doi.org/10.6084/m9.figshare.17032760.v1 Instructions for accessing these data: Navigate to the URL above using any web browser

## Value of the Data

• These data expand our current knowledge base and understanding of refractory high entropy alloys.
• These data can be incorporated with other datasets on similar alloy systems and/or used directly in analytics and machine learning workflows to develop new refractory alloy compositions with tailored properties.
• The experimental compressive ductility screening test method introduced here to generate ductility ranking data can be used directly or expanded for future high throughput screening efforts.

## 1 Data Description

All data is collected in a .csv file stored on the figshare platform [1]. The dataset contains 12 columns; the first 9 define alloy composition in atomic percentage (at%) and the last 3 are the predicted solidus, hardness, and ductility ranking collected on each alloy as described in the experimental design, materials, and methods section below. Each row represents a unique alloy and there are 86 total rows (alloys) in the datasheet. Table 1 contains explicit descriptions of each column.

**Table 1:** Description of columns in the refractory alloy dataset [1].

Column Header	Description
Hf(at%)	Atomic percentage of hafnium in the alloy
Mo(at%)	Atomic percentage of molybdenum in the alloy
Nb(at%)	Atomic percentage of niobium in the alloy
Re(at%)	Atomic percentage of rhenium in the alloy
Ru(at%)	Atomic percentage of ruthenium in the alloy
Ta(at%)	Atomic percentage of tantalum in the alloy
Ti(at%)	Atomic percentage of titanium in the alloy
W(at%)	Atomic percentage of tungsten in the alloy
Zr(at%)	Atomic percentage of zirconium in the alloy
Predicted Solidus (°C)	Solidus (melting point) of the alloy (°C) predicted using Thermo-Calc Software (TCHEA4 database)
Hardness (GPa)	Room temperature microhardness (GPa) of the alloy collected using a Vickers indent
Ductility Ranking (0–5)	Room temperature compressive ductility of the alloy on a scale from 0 to 5 as described in the experimental design, materials, and methods section (0 is extremely brittle; 5 is highly ductile)

## 2 Experimental Design, Materials and Methods

**Fig. 1:**
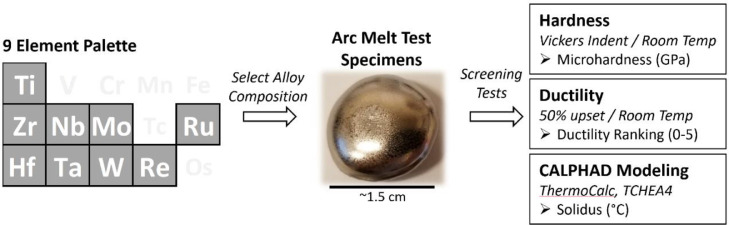
Overview of the current study including element design space, image of a typical arc melted test specimen, and details of screening tests and modelling output.

In this study we consider a 9-element alloy palette as shown in Fig. 1. After selecting an alloy composition, test specimens are produced via arc melting resulting in ∼1.5cm diameter coupons pictured in Fig. 1. Test specimens are subsequently sectioned and prepared for hardness and ductility testing. Thermodynamic (CALPHAD) modelling is also conducted to extract an estimated solidus (melting) temperature for each alloy. Details for each step in this screening process are included below. On average, we were able to process approximately 12 unique samples per week given equipment and personnel constraints – the dataset described here therefore represents about 2 months of applied work.

We anticipate that this dataset will be useful in conjunction with other sources [2] for researchers interested in RHEAs and refractory alloys in general. Our unique approach to screening room temperature ductility will also likely be of interest to the community as it is simple, flexible, fast, and sufficiently accurate to directionally guide development efforts. Quantifying ductility can be a challenge in high throughput operations but is a critical property to assess material manufacturability and damage tolerance.

New refractory alloy compositions are manufactured for experimental screening by arc melting. An appropriate amount of raw element stock is weighed and placed in individual divots on a water-cooled copper hearth in a Retec arc melter. After pulling vacuum and backfilling to 10psi Argon, a current of 600A is applied for 2 min at which point samples are flipped and remelted (again at 600A for 2min) a total of 4 times. Four unique chemistries can be processed (in individual divots) at a time in the setup used here. Specimens are nominally 1.5 cm in diameter and about 0.7 cm thick with a mass of ∼15g (composition dependent). Trace element content is kept as low as possible by using high purity raw element stock and keeping the arc melt chamber environment clean. Average trace element levels are O ≈ 0.042wt% and N ≈ 0.005wt%, primarily stemming from interstitials present in the raw stock. Total input mass and resulting melted specimen mass are tracked and flagged if mass change exceeds ±1%. In the event of excessive mass loss/gain a remelt is attempt during subsequent runs starting from fresh stock. In cases where a composition cannot be produced after two attempts without significant mass change the specimen is shelved and not analyzed further. Over the course of 100’s of refractory alloy melt attempts we routinely achieve approximately 85% yield using the method detailed above.

Specimens that are successfully arc melted are cut into multiple pieces using electrical discharge machining (EDM) for subsequent screening tests as outlined in Fig. 1. Hardness is measured on each sample using a standard Vickers microhardness indenter (Buehler Micromet 2100) at an applied load of 500g. Traditional metallographic preparation techniques are used to mount and polish specimens for indentation. Three indents are performed on each specimen and an average value is reported. In addition to measuring hardness, mounted and polished cross sections are also examined in an optical microscope to observe grain/phase structure and ensure no significant elemental un-melted particles remain in the alloy. This is done for every specimen to ensure thorough melting was achieved.

**Fig. 2:**
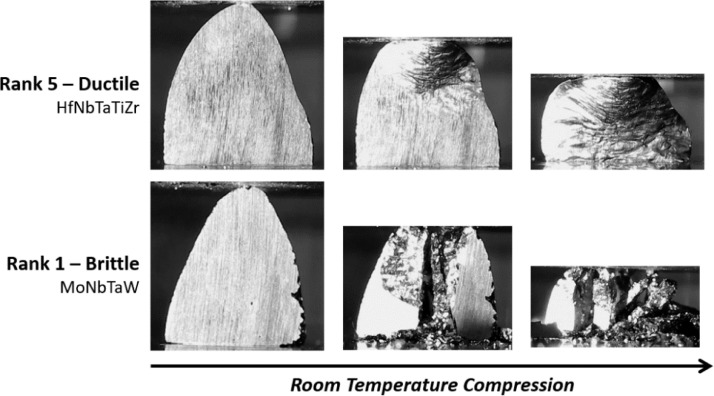
Video screenshots taken during the room temperature compression screening test showing typical ductile (rank 5) and brittle (rank 1) behaviour. Brittle samples crack early and tend to fracture into many pieces while ductile samples deform plastically with no cracking.

A separate section cut from arc melted samples is used to assess room temperature compressive ductility. To screen ductility, we compress sections in a standard servo-hydraulic test frame (MTS Landmark) at 2.5 mm/s displacement rate (corresponding to an initial strain rate on the order of 1 × 10^−3^ s^−1^) with an integrated video camera to observe deformation behavior. Traditional ductility metrics derived from stress-strain curves cannot be extracted here because we are using irregular shaped specimens; they are not machined to a standard shape. This is done intentionally to ensure the ductility screening test can be run on pace with arc melting and prevents excessive delays and costs associated with precise machining. While raw load-displacement data is collected during the screening tests, it is not of value due to the irregular and variable geometry of the screening test specimens. Instead, to interpret compressive ductility, we use a visual ranking approach based on video captured from each specimen to define ductility on a scale from 0 (low/brittle) to 5 (high/ductile). Rank 0 is reserved for specimens that are so inherently brittle they fracture during melting operations, handling, or EDM sectioning. These extremely brittle materials never make it to the compression test. Fig. 2 shows typical screenshots for rank 1 and 5 specimens from initial (time zero, no deformation), mid-test, and near-final compression to 50% upset. The materials shown in Fig. 2 are a notable ductile RHEA from the literature – equiatomic HfNbTaTiZr [3] – and a popular composition researched for high temperature strength – equiatomic MoNbTaW [4]. The ductile specimen (top row) deforms homogeneously, displaying significant bulk plasticity with no fracture. On the other hand, the brittle material (bottom row) fractures into many pieces almost immediately on the application of compressive strain. Following 50% upset the brittle sample has fractured into many small pieces; no distinguishable bulk plasticity is observed in Rank 1 specimens. Intermediate rankings of 2, 3, or 4 are assigned for behavior falling in between these two extremes. Ranking assignments become routine after observing the deformation behavior of multiple different specimens.

To test the repeatability of our ductility screening approach the alloys from Fig. 2 (HfNbTaTiZr [3] and MoNbTaW [4]) were melted and tested 3 times. The repeat tests were randomized and conducted blind – the test operator who assigns ductility rankings did not know the composition of the alloys. We also tested two commercial refractory alloys (not in the current dataset) known to have viable low temperature ductility – C103 and TZM. Ductility ranking results for all four alloys are presented in Table 2.

**Table 2:** Ductility rankings for 4 baseline alloys. HfNbTaTiZr and MoNbTaW were tested three times to assess repeatability; results for all three tests are included.

Alloy	Ductility Ranking (0–5)
HfNbTaTiZr	4, 4, 5
MoNbTaW	1, 1, 1
TZM	5
C103	5

The ductile RHEA HfNbTaTiZr scored ductility rankings of 4, 4, and 5 in three independent tests while the brittle RHEA MoNbTaW scored 1 for all tests. Commercial alloys TZM and C103 both scored a ductility ranking of 5. This is consistent with the expected behavior of these alloy systems and reinforces the validity of the ductility screening test used here. While not traditional, the ductility ranking approach is useful for directionally guiding alloy development projects in a high throughput framework. We are deliberately prioritizing *direction over perfection* for screening purposes. However, in cases where researchers may want to compare rankings with more common and standard ductility metrics we offer the following observations and suggestions. A ductility ranking of 0 indicates that the material has essentially no ductility and can be interpreted as such – failure with 0% plastic strain. On the other end of the spectrum, a ductility ranking of 5 indicates significant ductility on par with commercially viable alloys. These specimens survive 50% upset in compression at room temperature in our screening test without any sign of fracture. Therefore, an estimate of 50% in conventional terms (strain to failure) could be assumed and is in-line with room temperature elongation to failure measurements in C103 [5] and TZM [6]. For rankings in between 0 and 5 mapping to conventional ductility metrics becomes more subjective. With a ductility ranking of 1 the material survived melting and sectioning operations but fractured immediately on the application of compressive strain. Strain to failure of <1% is likely for these materials, and one could assign 0.1% as an estimate. At a ductility ranking of 2 early stage plastic deformation is observed on the application of strain followed closely by fracture; 1% strain to failure could be assumed for these materials. At ductility rankings of 3 and 4 the material displays observable plastic deformation over the course of testing but fractures before full 50% upset. Roughly interpolating, one could assign strains to failure of 10% and 20% for ductility rankings of 3 and 4, respectively. These assumptions are collected in Table 3, but the reader is cautioned that these are assumptions only and should not be taken as exact.

**Table 3:** Assumptions for room temperature strain to failure as measured in conventional mechanical testing for the ductility rankings defined in this work. Note that these are assumptions for reference only based on observed material behaviour and should not be taken as exact.

Ductility Ranking (0–5)	Strain to Failure Assumption (%)
0	0
1	0.1
2	1
3	10
4	20
5	50

In addition to hardness and low temperature ductility, alloy solidus (melting temperature) is included as a third parameter in the dataset. While effort was made to experimentally measure solidus in a high throughput workflow, the extreme temperatures – and therefore burden on the equipment (differential thermal analyzer) – made this test untenable. As a fallback we resort to CALPHAD assessment of each alloy and report a predicted solidus temperature. Thermo-Calc Software (TCHEA4 database) operated through TC-Python was used for these predictions.

## CRediT authorship contribution statement

**Andrew Detor:** Conceptualization, Methodology, Software, Validation, Writing – original draft, Visualization, Supervision, Project administration. **Scott Oppenheimer:** Methodology, Validation, Investigation, Resources. **Rebecca Casey:** Methodology, Validation, Investigation. **Cole Crawford:** Methodology, Investigation.

## Declaration of Competing Interest

The authors declare that they have no known competing financial interests or personal relationships that could have appeared to influence the work reported in this paper.

## Data Availability

GE_RefractoryAlloyScreeningDataset_FINAL.csv (Original data) (Figshare).

## References

[1] A. Detor, S. Oppenheimer, R. Casey, C. Crawford. ``GE_RefractoryAlloyScreeningDataset_FINAL.csv'', figshare. Dataset. 2021 10.6084/m9.figshare.17032760.v1.

[2] Borg C., Frey C., Moh J., Pollock T., Gorsse S., Miracle D., Senkov O., Meredig B., Saal J. (2020). Expanded dataset of mechanical properties and observed phases of multi-principal element alloys. Sci. Data.

[3] Senkov O., Semiatin S. (2015). Microstructure and properties of a refractory high-entropy alloy after cold working. J. Alloys Comp..

[4] Senkov O., Wilks G., Scott J., Miracle D. (2011). Mechanical properties of Nb_25_Mo_25_Ta_25_W_25_ and V_20_Nb_20_Mo_20_Ta_20_W_20_ refractory high entropy alloys. Intermetallics.

[5] ATI Wah Chang Nb/Nb Alloy C103, Alloy datasheet [Online]. Available: http://www.matweb.com/search/datasheet.aspx?matguid=ed50a3a07706450590669cedc7784150&ckck=1. (2022).

[6] Liu C., Inouye H. (1973).

